# Effects of Surface Charge of Amphiphilic Peptides on Peptide–Lipid Interactions in the Gas Phase and in Solution

**DOI:** 10.1021/acs.analchem.5c00283

**Published:** 2025-03-07

**Authors:** Til Kundlacz, Christian Schwieger, Carla Schmidt

**Affiliations:** † Institute of Chemistry, Martin Luther University Halle-Wittenberg, von-Danckelmann-Platz 4, 06120 Halle, Germany; ‡ Department of Chemistry—Biochemistry, Biocenter II, Johannes Gutenberg University Mainz, Hanns-Dieter-Hüsch-Weg 17, 55128 Mainz, Germany

## Abstract

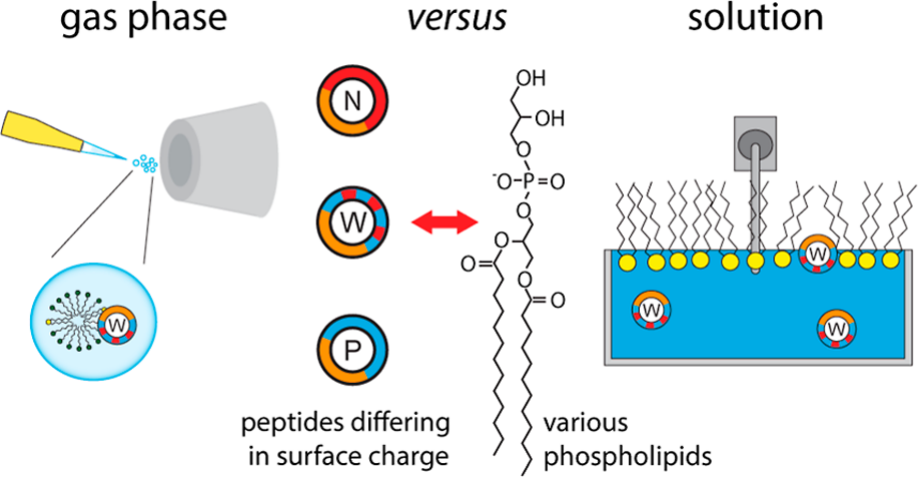

The interactions between peptides and lipids are fundamental for many biological processes. Therefore, exploring the noncovalent interactions that govern these interactions has become increasingly important. Native mass spectrometry is a valuable technique for the characterization of specific peptide–lipid interactions. However, native mass spectrometry requires the transfer of the analyte into the gas phase, and noncovalent interactions driven by the hydrophobic effect might be distorted. We, therefore, address the importance of electrostatic interactions for the formation of peptide–lipid interactions. For this, we make use of the amphipathic, antimicrobial peptide LL-37 as well as a positively and a negatively charged variant thereof and study binding of a variety of lipids by native mass spectrometry. We found that the surface charge of the peptides affects the transfer of stable peptide–lipid complexes into the gas phase and that the ionization mode is important to observe these interactions. We further compare our findings observed in the gas phase with interactions formed in solution between the peptides and lipid monolayers using a Langmuir film balance. The two approaches deliver comparable results and reveal a clear trend in the lipid preferences of all variants for those lipids with opposite charge. Notably, the unmodified wild-type peptide was more flexible in the formation of peptide–lipid interactions. We conclude that native mass spectrometry is indeed well-suited to explore the interactions between peptides and lipids and that electrostatic interactions as expressed by the surface charge of the peptides play an important role in the formation and stabilization of peptide–lipid interactions.

## Introduction

Protein–lipid and protein–membrane interactions are important for various cell functions including, for instance, enzymatic activity, membrane transport, or signaling and trafficking events. They are driven by an interplay of noncovalent interactions, including electrostatic interactions, van der Waals forces, and the hydrophobic effect. Depending on the protein’s structure and the binding mode to the lipid bilayer, the different types of noncovalent interactions contribute to its stable association. Importantly, the function of a membrane protein depends not only on correct protein folding but also on a specific lipid environment. The study of protein–lipid interactions, therefore, gained importance during the last decades. While classical structural techniques often fail to provide high-resolution structures of heterogeneous protein–lipid assemblies, other techniques identifying and characterizing the lipid environment of the proteins were introduced.

One such technique is native mass spectrometry (native MS),^1−6^ enabling the mass spectrometric analysis of intact protein–lipid complexes under nondenaturing conditions, thereby maintaining noncovalent interactions and resolving individual binding events.^7,8^ However, native MS requires the ionization and the transfer of the analyte from solution into the gas phase, where the hydrophobic effect is nonexistent and electrostatic interactions dominate.^9^ Accordingly, interactions caused by the hydrophobic effect cannot be stabilized in the gas phase and the question remains whether native MS is capable of accurately describing complexes formed by noncovalent interactions in solution. One approach that is commonly employed when analyzing protein–lipid interactions involves the transfer of lipids from mixed detergent–lipid micelles to soluble or membrane-associated proteins.^5,10,11^ Previously, we utilized this approach to analyze interactions between a model peptide, namely, the human antimicrobial peptide LL-37, and a variety of phospholipids.^12^ By varying the lipid classes and the length of the fatty acyl chains of the phospholipids, we systematically explored the electrostatic interactions of LL-37 with the lipid head groups as well as interactions with the fatty acyl chains of the lipids that are driven by the hydrophobic effect. We found that electrostatic interactions are stabilized in the gas phase and that interactions formed in solution are reflected by the intensity of the complexes during native MS measurements.

Here, we follow the same strategy as described above to investigate the effects of the peptide surface charge on peptide–lipid interactions with different lipids. For this, we again chose antimicrobial peptide LL-37 as a model peptide. Antimicrobial peptides are typically short peptides containing a net cationic charge and an amphipathic structure; these properties are essential for their selectivity for bacterial membranes, which contain a high proportion of anionic lipids.^13−15^ To study the effects of peptide surface charge, we designed a supercharged cationic (LL-37-pos) and a supercharged anionic (LL-37-neg) variant of LL-37 and compared their lipid interactions with those formed by the wild-type peptide (LL-37-wt). These supercharged variants include additional positively or negatively charged amino acids, altering their solution net charge from 6+ (LL-37-wt) to 14+ (LL-37-pos) or 14– (LL-37-neg). The three variants were investigated with respect to their interactions with negatively charged, zwitterionic, and positively charged lipids. However, as native MS analyses mostly reflect electrostatic interactions that are stable in the gas phase, we further studied the interactions that are formed between the LL-37 variants and lipid monolayers in solution. For this, we employed an adsorption film balance and assembled Langmuir monolayers that are composed of different phospholipids at the air–water interface. Langmuir monolayers represent one leaflet of a phospholipid bilayer and are a widely used as membrane model systems.^16,17^ Importantly, interactions between peptides (or proteins) and the monolayers involve hydrophobic as well as electrostatic interactions, therefore allowing the formation of natural binding interfaces including insertion of the peptides into the membrane. Due to their amphipathic structure and natural membrane binding propensity, antimicrobial peptides such as LL-37 are well-suited model peptides to investigate peptide–membrane interactions as well as peptide insertion into the membranes using an adsorption film balance.^18−21^

Using native MS, we first determine the lipid preferences of the three LL-37 variants (i.e., LL-37-wt, LL-37-pos, and LL-37-neg) in the gas phase. While lipid binding of the cationic variants LL-37-wt and LL-37-pos was successfully assessed in positive ion mode, which is commonly employed for proteins and peptides, the analysis of the anionic variant (LL-37-neg) required the application of the negative ion mode, demonstrating that the ionization affects lipid binding and stabilization of peptide–lipid complexes in the gas phase. Importantly, by making use of an adsorption film balance, lipid binding preferences of the variants in solution were explored and compared with the results obtained in the gas phase. Accordingly, gas phase and solution measurements correlate well when considering the surface charge of the peptides and the required ion modes during native MS experiments.

## Experimental Methods

### Materials

1-*O*-(*n*-Octyl)-tetraethylene glycol (C8E4) was purchased from Glycon Biochem (Luckenwalde, Germany). 7.5 M ammonium acetate (AmAc) solution (7.5 M) and PBS tablets were purchased from Sigma-Aldrich (St. Louis, USA). Ammonium bicarbonate (≥99%) was purchased from Carl Roth (Karlsruhe, Germany). Chloroform (HPLC grade) was purchased from Alfa Aesar (Haverhill, USA). Methanol (LC/MS grade) and acetic acid (LC/MS grade) were purchased from Fisher Scientific (Hampton, USA).

Human LL-37 (trifluoroacetate salt, ≥95% purity, Sigma-Aldrich (St. Louis, USA)) was dissolved in phosphate-buffered saline (PBS) from Sigma-Aldrich (St. Louis, USA) and stored at −20 °C. A positively (LL-37-pos) and a negatively (LL-37-neg) charged variant of LL-37 were obtained from Thermo Scientific Custom Peptide synthesis service (Waltham, USA) as lyophilized trifluoroacetate salts: LL-37-pos (amino acid sequence: LLGKFFRKSKKKIGKKWKRIVQRIKKFLRNLVPRTES) and LL-37-neg (amino acid sequence: LLGDFFEESEEEIGEEWEEIVQEIEDFLENLVPRTES). Note that a phenylalanine at position 17 was substituted for tryptophan to provide the variants with spectroscopic properties. Notably, single phenylalanine to tryptophan LL-37 mutants display similar behavior to the wild-type.^20^ LL-37-pos was dissolved in 25% (v/v) acetic acid, further diluted with water to a final peptide concentration of 1 mg/mL, and stored at −20 °C. LL-37-neg was dissolved with 0.1 M ammonium bicarbonate (Carl Roth GmbH, Karlsruhe, Germany), further diluted with water to a final peptide concentration of 1 mg/mL, and stored at −20 °C.

1,2-Dimyristoyl-*sn*-glycero-3-phospho-(1′-rac-glycerol) (PG 14:0/14:0), 1,2-dimyristoyl-*sn*-glycero-3-phospho-l-serine (PS 14:0/14:0), 1,2-dimyristoyl-*sn*-glycero-3-phosphoethanolamine (PE 14:0/14:0), 1,2-dimyristoyl-*sn*-glycero-3-phosphocholine (PC 14:0/14:0), 1,2-dimyristoyl-*sn*-glycero-3-phosphate (PA 14:0/14:0), and 1,2-dimyristoyl-3-trimethylammonium-propane (TAP 14:0/14:0) were purchased from Avanti Polar Lipids (Alabaster, USA). The lipids were dissolved in pure chloroform or 2:1 chloroform:methanol (v/v) and stored in aliquots. For this, the solvent was evaporated under a nitrogen stream, and dried lipids were overlaid with argon. Aliquots were stored at −20 °C. The lipid content was verified by photometric phosphate analysis.^22^ An overview of the lipid structures is given in Figure S1.

### Preparation of Mixed Detergent–Lipid Micelles

For transfer of lipids to peptide variants during electrospray ionization (ESI), mixed detergent–lipid micelles were prepared as follows: dried lipids were resuspended in 200 mM AmAc, pH 7.5 containing 0.5% (w/v) C8E4 and sonicated for 30 min at 60 °C. For complete solubilization of TAP 14:0/14:0, sonication was performed at 70 °C followed by two freeze/thaw cycles.

### Dynamic Light Scattering

The mean hydrodynamic diameter of detergent–lipid micelles and C8E4 micelles was determined using a Litesizer 500 particle size analyzer (Anton Paar, Graz, Austria). For this, 100 μL of a detergent–lipid micelle suspension were analyzed in a 3 × 3 mm ultra-microcuvette (Hellma Analytics, Müllheim, Germany). The particles were irradiated with a semiconductor laser diode at 658 nm by employing the following instrument settings: measuring angle, side scatter (90°); temperature, 25 °C; measurement time, automatic; filter, automatic; focus, automatic; material, phospholipids. The mean hydrodynamic diameter was determined from size distribution histograms using Kalliope (Anton Paar, Graz, Austria).

### Circular Dichroism Spectroscopy

For UV–vis circular dichroism (CD) spectroscopy, 50 μL of a 1 mg/mL peptide solution in PBS and 200 mM AmAc in the presence and absence of 0.5% (w/v) C8E4 were analyzed in a 0.1 mm quartz cuvette at 20 °C using a J-810 spectropolarimeter (JASCO, Groβ-Umstadt, Germany). The following instrument parameters were applied: wavelength, 190–240 nm; scanning mode, continued; scan number, 64 scans; scan speed, 50 nm/min; response, 1 s; data pitch, 1 nm. The raw data was reduced to data points at HT voltage below 600 V as the signal-to-noise ratio is lower at high dynode voltages. CD spectra were smoothed using a binomial filter, and a reference spectrum was subtracted using the Spectra Manager software (JASCO). The ellipticity was converted to mean residue ellipticity (Δε) as described previously.^23^

### Sample Preparation for Native MS

LL-37 variants were transferred to 200 mM AmAc using Micro Bio-Spin P6-6 gel columns (Bio-Rad, Hercules, USA) according to the manufacturer’s instructions. The peptide concentration was subsequently determined using the Bradford assay^24^ (LL-37-wt) or by UV–vis spectroscopy at 280 nm (LL-37-pos and LL-37-neg). Prior to native MS analysis, 20 μM of the LL-37 variants was mixed with the detergent–lipid micelles to final concentrations of 25 μM lipid and 0.5% (w/v) C8E4.

### Native MS

All measurements were performed using a Q-TOF Ultima mass spectrometer (Waters, Wilmslow, UK) modified for native MS.^25^ For each individual measurement, 3 μL of the sample were loaded into a gold-coated borosilicate emitter needle produced in-house.^26^ The analysis was performed in positive or negative ion mode.

Instrument settings for positive ion mode were as follows: capillary voltage, 1.7 kV; capillary temperature, 80 °C; cone voltage, 35 V; collisional voltage, 30 V; and RF lens voltage, 80 V. Four replicates were performed for each measurement.

Instrument settings for negative ion mode: capillary voltage, 1.0 kV; capillary temperature, 80 °C; cone voltage, 35 V; collisional voltage, 30 V; and RF lens voltage, 80 V. Four replicates were performed for the interaction of LL-37-neg with TAP 14:0/14:0 and three replicates for the interaction with PC 14:0/14:0 and PG 14:0/14:0, respectively.

### Data Analysis

The UniDec^27^ software was used for deconvolution of unprocessed mass spectra. The following settings were employed: *m*/*z* range, 750 to 4600; Gaussian smoothing, 20; background subtraction, 20; charge range, 1 to 8; mass range, 4400 to 6900 Da; peak full width half-maximum, ∼3.4. The intensity (termed “height” in UniDec settings) of selected peaks was extracted after normalization of the mass spectra to the base peak. Extracted peak intensities of all charge states of the peptide–lipid complexes were summed and divided by the extracted peak intensity of the total peptide monomer peaks, yielding relative abundances of the peptide–lipid complexes.

For visualization of mass spectra, raw data were processed using MassLynx v4.1 (Waters, Wilmslow, UK). At least 70 scans were combined and smoothed twice with a smooth window of 20 using the Savitzky–Golay filter^28^ followed by background subtraction applying a 30% reduction under the curve with a polynomial order of 3 and a tolerance of 0.01.

### Film Balance Measurements

Film balance experiments were performed using a DeltaPi-4x Langmuir Tensiometer (Kibron, Helsinki, Finland). PBS was used as the aqueous phase for all experiments (2.1 mL of PBS per trough). All experiments were performed at 20 °C; the temperature was controlled by using an external circulating water bath. During the measurements, the film balance was covered with an acrylic glass cover to avoid dust accumulation, as well as evaporation of the subphase. Small water reservoirs under the acrylic glass cover further reduced sample evaporation during the measurements. The subphases were gently stirred throughout the measurements.

Before the analysis, the instrument was calibrated against the known surface pressure (π) of water at 20 °C (72.8 mN/m). Subsequently, the surface pressure of the subphase was measured for at least 10 min to detect potential surface contaminants. The peptide samples were prepared as follows: LL-37 variants were transferred to PBS using 3 kDa MWCO Amicon Ultra Centrifugal Filters (Merck Millipore, Billerica, USA). The protein concentration was determined using the Bradford assay^24^ (LL-37-wt) or by UV–vis spectroscopy at 280 nm (LL-37-pos and LL-37-neg).

#### Determination of the Surface Activity

To determine the surface activity of the individual LL-37 variants as well as appropriate peptide concentrations for monolayer adsorption studies, the adsorption at the air–water interface was analyzed. For this, peptide concentrations of 100–900 nM (LL-37-wt), 25–500 nM (LL-37-pos), and 25–750 nM (LL-37-neg) were injected into the subphase, and the increase in surface pressure (Δπ), caused by accumulation of the peptides at the air–water interface, was measured as a function of time for 3–6 h. For data analysis, Δπ was plotted against the peptide bulk concentration and fitted using an exponential association function [*y* = *y*_0_ + *A*_1_ (1 – e^–*x*/*t*1^) + *A*_2_ (1 – e^–*x*/*t*2^)]. Δπ increases with the peptide concentration until a plateau, indicating surface saturation, is reached. The peptide concentration for monolayer adsorption studies was chosen such that saturation is assured.

#### Adsorption of Peptides to Lipid Monolayers

To determine the adsorption of LL-37 variants to a lipid monolayer, lipid monolayers were prepared by gradually spreading different lipids (PC 14:0/14:0, PG 14:0/14:0, or TAP 14:0/14:0) dissolved at 0.1 mg/mL in chloroform or 2:1 chloroform:methanol (v/v) at the air–water interface until the desired initial surface pressure (π_0_) is reached. The lipid film was then equilibrated for approximately 30 min. Subsequently, the peptides were injected into the subphase underneath the lipid film. The final peptide concentration in the subphase was 400 nM for all variants. After peptide injection, the surface pressure was measured as a function of time for 4 to 8 h until the surface pressure reached an equilibrium value (π_eq_). The change in surface pressure (Δπ), caused by insertion of the peptides into the lipid monolayer, is calculated as Δπ = π_eq_ – π_0_. For each LL-37 variant and each lipid monolayer, several measurements at different initial surface pressures π_0_ were performed. For data analysis, Δπ was plotted against π_0_ and fitted with a linear function Δπ = *A*·π_0_ + *B*. The maximum insertion pressure (MIP) was determined by extrapolating the plot of Δπ as a function of π_0_ to Δπ = 0. The MIP equals the intercept of the linear plot with the *x* axis. Error bars of the MIP values were calculated using the Binding Parameter Calculator software.^29^

## Results and Discussion

### Generation and Characterization of Surface-Charge Variants

In a previous study, we explored the noncovalent interactions of a peptide with lipids of different electrostatic and hydrophobic properties to determine whether observations in the gas phase reflect interactions that are formed in solution.^12^ In that study, we showed that electrostatic interactions formed between the lipid head groups and the peptide in solution are stabilized in the gas phase. However, these experiments only addressed the effects of different lipid head groups and the question remains whether the surface charge of a peptide affects the formation of peptide–lipid complexes in solution and in the gas phase. We, therefore, set out to investigate the influence of the surface charge of peptides on peptide–lipid interactions formed in solution and observed in the gas phase.

**Figure 1:**
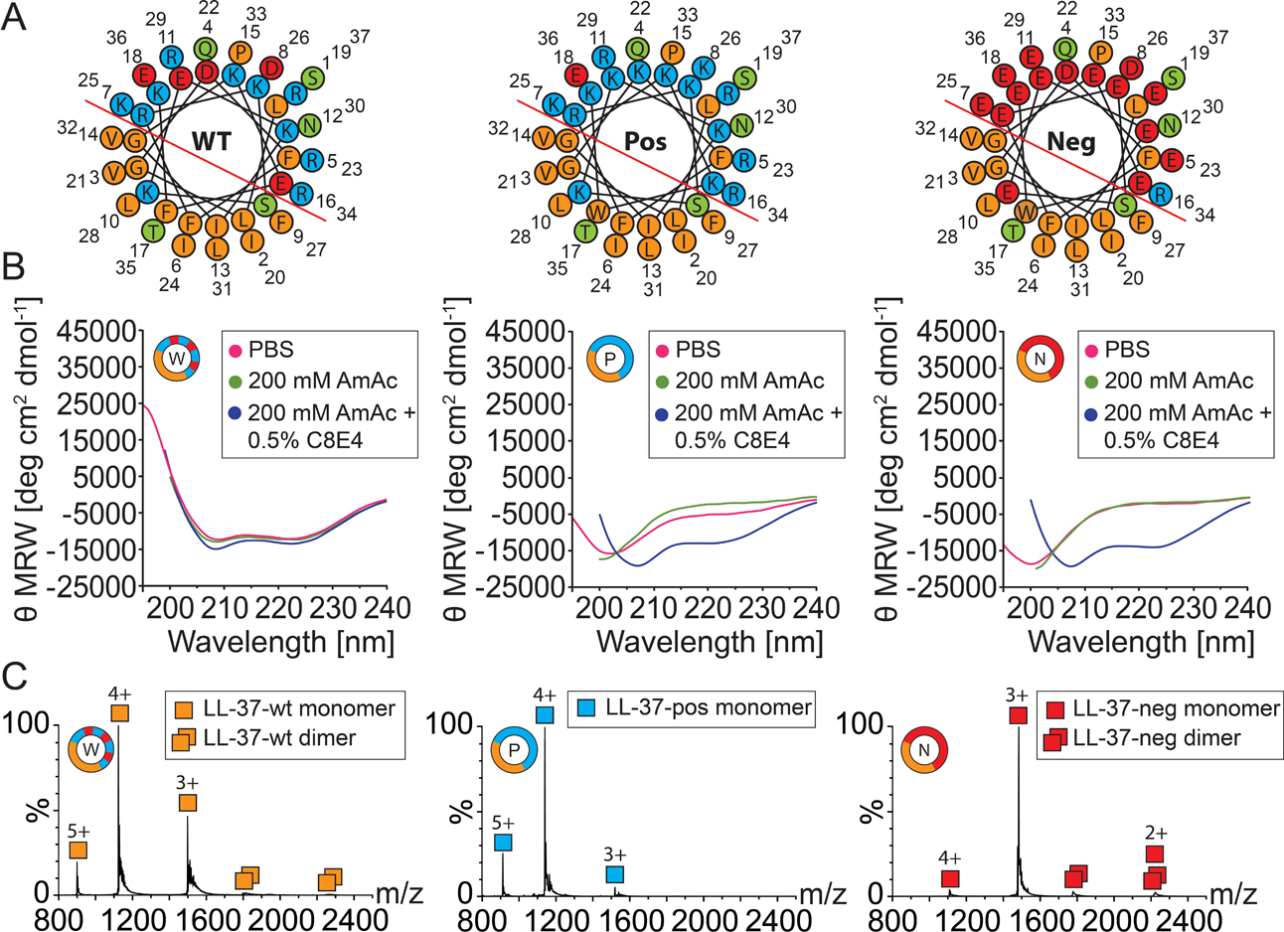
Characterization of LL-37 variants. (A) Helical wheel projection of LL-37-wt (lhs), LL-37-pos (middle), and LL-37-neg (rhs) created with HeliQuest.^51^ Hydrophobic (orange), basic (blue), acidic (red), and polar uncharged residues (green) are shown. The hydrophobic–hydrophilic interface is indicated (red line). (B) CD spectra of LL-37-wt (lhs), LL-37-pos (middle), and LL-37-neg (rhs) acquired in PBS (pink), 200 mM AmAc (green), and 200 mM AmAc, 0.5% (w/v) C8E4 (blue). (C) Native mass spectra of 10 μM LL-37-wt (lhs), LL-37-pos (middle), and LL-37-neg (rhs) in 200 mM AmAc. Charge states as well as monomeric (squares) and dimeric (two squares) species are assigned. For masses of determined species, see Table S2.

For this, we designed a systematic study using antimicrobial peptide LL-37 as a model peptide. According to its function, LL-37 forms an amphipathic helix required for membrane integration during antimicrobial defense.^13,30,31^ This mechanism involves interactions between the hydrophobic interface of the amphipathic helix and the fatty acyl chains of the phospholipids as well as electrostatic interactions between the hydrophilic interface of LL-37 and the lipid head groups. To study the effects of surface charge on these interactions, we designed a positively and negatively charged variant of LL-37 in addition to the wild-type peptide. Accordingly, aspartate and glutamate residues of LL-37 residing in the amphipathic helix were replaced by lysine residues (positively charged variant, LL-37-pos) or lysine and arginine residues of the LL-37 sequence were substituted with glutamate residues (negatively charged variant, LL-37-neg). By replacing a multitude of residues rather than individual amino acids, we generated supercharged variants of LL-37 that differed significantly in their solution net charge, allowing us to attribute observed effects to differences in surface charge of the peptides. Importantly, while the physiochemical properties of the three LL-37 variants (i.e., LL-37-wt, LL-37-pos, and LL-37-neg) differ significantly (Table S1), the amphipathic structure of LL-37 consisting of a hydrophobic and a hydrophilic interface is maintained as shown in the helical wheel projections (Figure 1A).

To investigate the influence of the modifications onto helix formation of the LL-37 variants, the secondary structure content of both LL-37-pos and LL-37-neg was assessed by CD spectroscopy and compared with the wild-type peptide (Figure 1B). To mimic the experimental conditions employed in native MS and in film balance experiments, we used PBS or 200 mM AmAc with and without 0.5% (w/v) C8E4. The CD spectrum of LL-37-wt was acquired in the presence of PBS and revealed local minima at 208 and 222 nm, which are characteristic for alpha helical structures.^32^ In contrast, LL-37-pos and LL-37-neg showed a local minimum at 203 nm indicating that both variants are unstructured in PBS.^33^ Similarly, in the presence of 200 mM AmAc, LL-37-wt adopts a helical conformation, while LL-37-pos and LL-37-neg are unfolded. Notably, when 0.5% (w/v) C8E4 was added to 200 mM AmAc, all LL-37 variants adopted an α helix indicating that the C8E4 detergent induces a transition of LL-37-pos and LL-37-neg from an unstructured to an α-helical conformation; accordingly, the helical content of LL-37-wt increased in the presence of C8E4. This is in agreement with previous findings showing that the formation of alpha helices in a hydrophobic environment has been described for many antimicrobial peptides.^34−37^ Interestingly, the ionic strength of PBS or 200 mM AmAc is not sufficient to induce structure formation of LL-37-pos and LL-37-neg as proposed for LL-37-wt earlier.^13^ Importantly, buffer conditions as employed during native MS induce the formation of an α helix, guaranteeing that observed effects are not an effect of structural differences between the variants. In addition, helix formation is a prerequisite for biological function of the peptides^13^ and functional activity of the peptides is, therefore, anticipated.

Next, we assessed the ionization behavior of the three variants during native MS in positive ion mode, as commonly employed for proteins and peptides (Figure 1C). The acquired mass spectra showed three charge states for all variants. For LL-37-wt and LL-37-pos, charge states ranging from 3+ to 5+ corresponding to the monomeric peptide were observed. LL-37-neg showed a small shift toward lower charge states resulting in charge states from 2+ to 4+. In all cases, the monomeric peak distribution was predominant; LL-37-wt and LL-37-neg showed a minor distribution (<5%) of the dimeric peptide. Note that the charge states observed in these measurements do not correlate with the charges of the peptides in solution (Table S1); this phenomenon was previously discussed in detail.^38−40^ As the ionization of peptides is best described by the charged residue model,^41^ the number of acquired charges of peptides correlates with the Rayleigh charge of the ESI droplet and, therefore, the surface-accessible area of the peptides.^42−45^ Accordingly, LL-37-wt and LL-37-pos contain more potential protonation sites (i.e., basic amino acid residues) than the number of charges observed by native MS,^46^ and a similar charge state distribution was observed for the two variants. The small shift in the charge state distribution observed for LL-37-neg might be explained by the fact that a lack of protonation sites has only minor effects on the observed charge states^47,48^ and, therefore, positive charges during ionization are less stabilized (resulting in a reduction of only one acquired charge). Accordingly, in positive ion mode, carboxyl groups do not contribute to the charges acquired during ESI as they are neutralized by proton transfer during the ionization process.^49,50^ In addition, LL-37-neg might adopt a conformation different from that of LL-37-wt and LL-37-pos.

### Effects of Surface Charge on Peptide–Lipid Interactions in the Gas Phase

To assess protein–lipid interactions in the gas phase, we carefully optimized the instrument settings, including cone and collisional voltages. Accordingly, due to adduct formation of C8E4, collisional voltages below 30 V were not applied. The same optimized settings were employed for native MS measurements of all three variants. In addition, as the position of the ESI emitter significantly affects the ionization of analytes,^52^ the position of the ESI emitter was maintained in a similar position in relation to the cone in all measurements.

Before transferring lipids to the LL-37 variants, we first studied the effect of the C8E4 detergent on their ionization properties (Figure S2). For this, the three variants were mixed with C8E4 detergent micelles and subsequently analyzed by native MS. As reported previously,^53,54^ charge reduction was observed in the presence of C8E4. Accordingly, for LL-37-wt and LL-37-neg, average charge states of 3.1+ (LL-37-wt) and 2.3+ (LL-37-neg) were observed in comparison to average charge states of 3.8+ (LL-37-wt) and 3.0+ (LL-37-neg) in the absence of C8E4 (Figures 1 and S2). On the contrary, this effect was not as obvious for LL-37-pos, which showed similar average charge state in the absence (4.2+) and presence (3.9+) of C8E4. Presumably, LL-37-pos stabilizes positive charges on the surface of the ESI droplet; due to its high gas phase basicity, charge reducing effects of C8E4 are minimized. This is in agreement with a recent study proposing that proteins with a higher gas phase basicity, i.e., proteins that contain more basic residues with high-affinity protonation sites, are more resistant to charge reduction.^47^ Interestingly, in the presence of this charge-reducing detergent, the three variants reflect the expected distribution of charges with the most intense charge state observed for LL-37-pos and the lowest for LL-37-neg.

Next, we analyzed the interactions of the three LL-37 variants with three negatively charged phospholipids (PA 14:0/14:0, PG 14:0/14:0, and PS 14:0/14:0) and two zwitterionic phospholipids (PC 14:0/14:0 and PE 14:0/14:0) as well as one positively charged, non-natural lipid analogue (TAP 14:0/14:0) by native MS (see Figure S1 for an overview of the lipid structures). For transfer of lipids to the peptides, detergent–lipid micelles were prepared (see Section Experimental Methods), mixed with the LL-37 variants and subsequently subjected to ESI and native MS. Note that detergent–lipid micelles including TAP showed a typical hydrodynamic diameter of approximately 6 nm comparable with C8E4 micelles (Figure S3). The acquired mass spectra revealed interactions of all LL-37 variants with the three negatively charged phospholipids (Figure S4); charge state distributions corresponding in mass to the LL-37 variant with up to three (LL-37-wt and LL-37-pos) or two (LL-37-neg) associated lipids were observed. The mass spectra acquired with the zwitterionic phospholipids and the cationic lipid analogue (Figure S5) revealed the binding of up to two zwitterionic lipids to all variants. Interestingly, binding of TAP 14:0/14:0 was observed only to LL-37-neg, while LL-37-wt and LL-37-pos did not bind this lipid. In summary, the complexes formed between negatively charged lipids and LL-37-wt and LL-37-pos showed higher intensities and higher numbers of associated lipids (up to three) than those of the complexes formed with zwitterionic lipids (up to two). In contrast, intensities and numbers of associated lipids observed for peptide–lipid complexes including LL-37-neg were low for all lipids employed.

**Figure 2:**
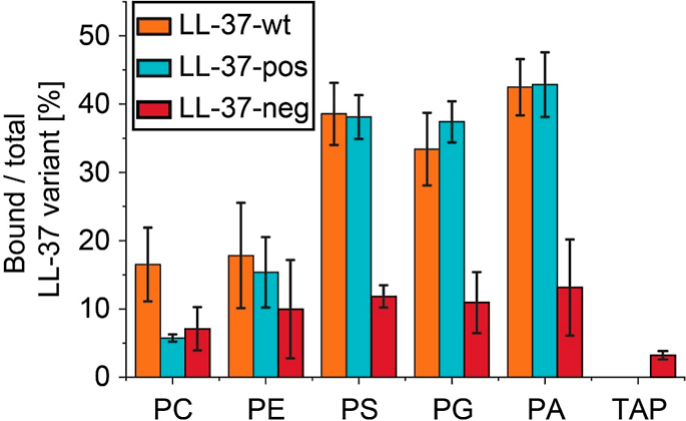
Interactions of LL-37 variants with different glycerophospholipids and TAP. Relative abundances of the complexes formed between LL-37-wt (orange), LL-37-pos (cyan), or LL-37-neg (red) and PC 14:0/14:0, PE 14:0/14:0, PS 14:0/14:0, PG 14:0/14:0, PA 14:0/14:0, and the lipid analogue TAP 14:0/14:0 were determined and compared. Error bars show the standard deviations between replicates (*n* = 4).

In order to determine lipid binding preferences of the three LL-37 variants, we determined and compared relative abundances of peptide–lipid complexes as described (Section Experimental Methods and Figure 2). The relative abundances of detected peptide–lipid complexes for LL-37-wt and LL-37-pos were mostly comparable with the exception of peptide–lipid complexes containing PC 14:0/14:0, which showed higher abundances for LL-37-wt. In detail, complexes containing negatively charged phospholipids (i.e., PS 14:0/14:0, PG 14:0/14:0, and PA 14:0/14:0) showed relative intensities of up to 40%, while the intensities of complexes containing zwitterionic lipids (i.e., PC 14:0/14:0 and PE 14:0/14:0) were lower than 20%. Complexes containing the cationic lipid analogue TAP 14:0/14:0 were not detected. These results are in agreement with previous studies demonstrating the preference of LL-37-wt for negatively charged glycerophospholipids.^21,55−57^ For LL-37-neg, relative abundances of peptide–lipid complexes were generally low (<15%). Despite its negative charge in solution, binding of the cationic TAP 14:0/14:0 was less abundant. Notably, slightly higher intensities were determined for complexes containing negatively charged lipids when compared to zwitterionic lipids.

Nonetheless, we observed the following trend of lipid preferences for all LL-37 variants: negatively charged > zwitterionic > positively charged. Considering the different electrostatic properties of the LL-37 variants, these results were surprising. We, therefore, hypothesize that the observed peptide–lipid interactions are determined by the following factors: (i) The transfer of lipids from detergent–lipid micelles does not reflect conditions of a lipid bilayer such as chain packing, membrane curvature, or lipid–lipid interactions. In addition, the transfer efficiency of the lipids might also differ. (ii) Interactions with the accessible negative charge of the phosphate group in phospholipids likely enhance the binding to LL-37-wt and LL-37-pos. (iii) During native MS measurements, interactions with negatively charged lipids are likely stabilized in positive ion mode. Presumably, the ionization of cationic and zwitterionic lipids is more efficient and dissociation of these lipids is facilitated; accordingly, their ionization is best explained by the ion evaporation model. (iv) The neutralization of carboxyl groups by proton transfer during ESI in positive ion mode^49,50^ results in a lack of negative charges that stabilize interactions with positively charged functional groups in the gas phase.

### Exploring the Influence of Ionization Mode on Observed Peptide–Lipid Interactions

Hypothesizing that binding of negatively charged lipids is favored in positive ion mode, we next analyzed peptide–lipid interactions of the LL-37 variants in negative ion mode to uncover potential differences in the binding behavior. For this, we first optimized the MS conditions for measurements in negative ion mode. However, while the analysis of LL-37-neg revealed a narrow charge state distribution and a low degree of adduct formation, we were not able to analyze LL-37-wt and LL-37-pos in negative ion mode due to low ionization. We, therefore, only proceeded by fine-tuning instrument parameters for the analysis of LL-37-neg and its complexes.

Applying the optimized instrument parameters for negative ion mode, we analyzed LL-37-neg in 200 mM AmAc in the presence and absence of 0.5% (w/v) C8E4 as well as with three lipids, namely, zwitterionic PC 14:0/14:0, negatively charged PG 14:0/14:0, and the cationic TAP 14:0/14:0. Native mass spectra revealed a shift in the charge state distribution to higher charge states in the presence of C8E4 (Figure S5). Remarkably, despite similar cone and collision voltages, we did not observe unspecific fragmentation of LL-37-neg in negative ion mode, indicating stabilization of the peptide similar to membrane proteins as described before.^58^ These measurements revealed binding of PC 14:0/14:0 and TAP 14:0/14:0 to LL-37-neg with a maximum of two associated lipids (Figure S6). Interestingly, the binding of PG 14:0/14:0 to LL-37-neg was not observed.

**Figure 3:**
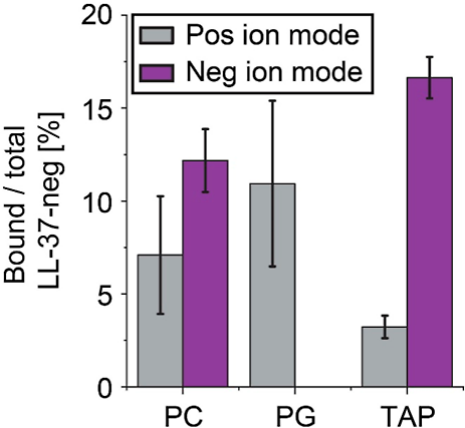
Interactions of LL-37-neg with lipids analyzed by native MS in negative ion mode. The relative abundance of LL-37-neg–lipid complexes containing PC 14:0/14:0, PG 14:0/14:0, or TAP 14:0/14:0 is given for positive (gray) and negative (purple) ion modes. Relative abundances were obtained from native mass spectra acquired in positive (Figures S3 and S4) and negative (Figure S5) ion modes. Error bars show the standard deviations between replicates (*n* = 3 for PC and PG negative ion mode; *n* = 4 for TAP in negative ion mode and PC, PG, and TAP in positive ion mode).

To compare binding of lipids to LL-37-neg in positive and negative ion modes, we determined the relative abundances of the peptide–lipid complexes for LL-37-neg with all of the tested lipids (Figure 3). In negative ion mode, we observed higher abundances for complexes containing PC 14:0/14:0 (approximately 12%) and TAP 14:0/14:0 (approximately 17%) compared to abundances observed in positive ion mode (approximately 7 and 3%, respectively). Importantly, binding of PG 14:0/14:0 was not detected in negative ion mode, while an abundance of LL-37-neg-PG complexes of approximately 11% was obtained in positive ion mode. The differences observed in relative abundances of the LL-37-neg–lipid complexes indicate that the ion mode has major influences on the lipid preferences of LL-37-neg. Neutralization of negative charges during ESI in positive ion mode might reduce interactions with zwitterionic lipids and the positively charged lipid analogue in the gas phase. Accordingly, LL-37-neg-lipid complexes are not sufficiently stabilized and, therefore, not observed in the mass spectra. Furthermore, the ionization efficiency of the peptide and the lipids in the different ion modes might also influence the observed interactions (see above); however, it is challenging to determine the degree of unspecific association and dissociation in these measurements.

### Investigating the Surface Activity of LL-37 Variants

Having investigated the lipid preferences of the LL-37 variants in the gas phase by native MS, we aimed to investigate the interactions of the variants with lipids in solution. For this, we made use of a Langmuir film balance and studied the adsorption of the LL-37 variants to single component lipid monolayers in solution. Lipid monolayers mimic one leaflet of a phospholipid bilayer and, therefore, represent a suitable model system for analyzing peptide–membrane interactions including binding and insertion of the peptides. These interactions are observed as changes in the surface pressure of the lipid film, i.e., peptide insertion leads to an increase in surface pressure.^59,60^

**Figure 4:**
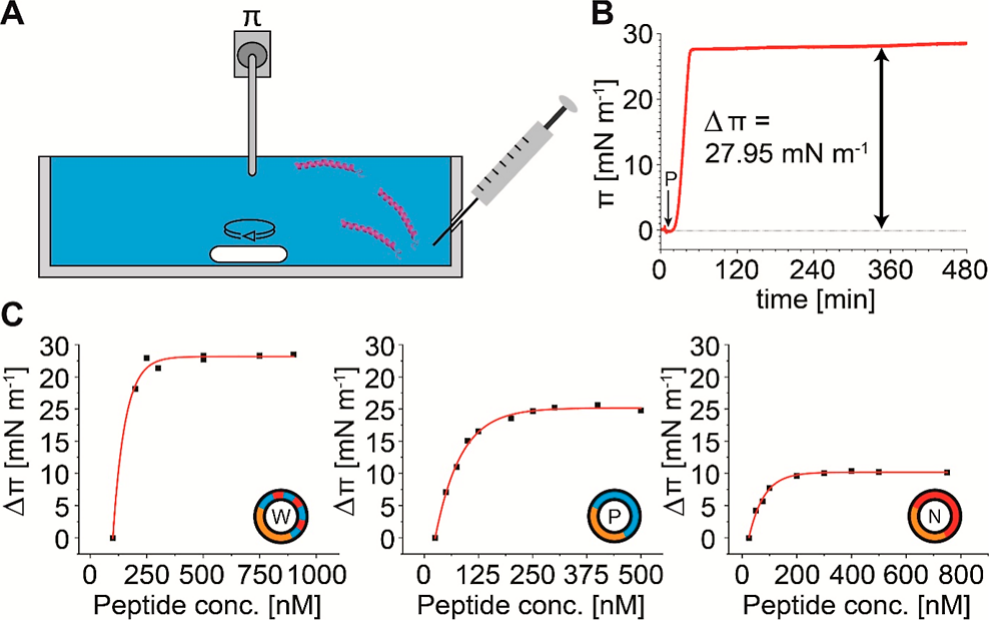
Surface activity of the three LL-37 variants. (A) Schematic of the experimental setup. Peptides were directly injected into the subphase (PBS). The increase in surface pressure resulting from the adsorption of peptides at the air–water interface is measured by a metal probe. (B) The surface pressure is plotted against time for the adsorption of 250 nM of LL-37-wt as an example. The time point of peptide injection (*P*) and the increase in surface pressure (Δπ) are indicated. (C) Δπ was plotted against the peptide concentration of LL-37-wt (lhs), LL-37-pos (middle), and LL-37-neg (rhs).

Before exploring the interactions of the LL-37 variants with different lipid monolayers, we first studied their adsorption at the air–water interface (i.e., without lipid film) as a function of peptide concentration. For this, different peptide concentrations were directly injected into the subphase consisting of PBS buffer (see Figure 4A for the experimental setup) and the adsorption of the peptide to the air–water interface was determined by monitoring the increase in surface pressure (π) (Figure 4B). Adsorption of the peptide at the air–water interface proceeds until reaching a plateau of equilibrium surface pressure (π_eq_). The increase in surface pressure (Δπ) is determined for the individual LL-37 variants at varying concentrations from these plateaus (Figure S7). To determine the surface activity of the individual LL-37 variants, Δπ is plotted against the peptide concentration (Figure 4C). An increase in Δπ with increasing peptide concentration was observed for all of the LL-37 variants. At peptide concentrations of approximately 350 nM (LL-37-wt) or 300 nM (LL-37-pos and LL-37-neg), Δπ reaches its maximum, indicating the subphase concentrations at which the air–water interface is saturated with adsorbed peptide. This pressure is related to the surface activity of the peptides. Following this approach, we determined surface saturation pressures of approximately 28 mN/m for LL-37-wt, 20 mN/m for LL-37-pos, and 10 mN/m for LL-37-neg, indicating that LL-37-wt has the highest and LL-37-neg the lowest surface activity. To test weather surface activity correlates with the hydrophobicity of peptides, the GRAVY score^61,62^ of the peptides was calculated (Table S1). In contrast to the observed surface activities (LL-37-neg < LL-37-pos < LL-37-wt), the GRAVY, describing the hydrophobicity of the peptides, increases in the order LL-37-pos < LL-37-wt < LL-37-neg. Therefore, the surface activity is not only defined by the net hydrophobicity of the peptides but also influenced by other factors. For instance, a correct secondary structure formation is required for the peptides to obtain their amphipathic properties.^63^ Accordingly, the secondary structure analysis of the LL-37 variants in PBS by CD spectroscopy (see above, Figure 1B) revealed that only LL-37-wt formed an α helix in PBS, while LL-37-pos and LL-37-neg were unfolded in solution. The lack of a secondary structure in the absence of a detergent might explain the low surface activities observed for LL-37-pos and LL-37-neg. Notably, many amphipathic peptides are known to fold only in a hydrophobic environment;^20,34−37^ we, therefore, expect the LL-37 variants to adopt an α-helical conformation in the presence of lipid monolayers. Nonetheless, based on the described experiments, we selected a peptide concentration of 400 nM for exploring the interactions of the LL-37 variants with lipid monolayers. This assures that the subphase concentration does not limit the potential adsorption to the lipid monolayers, and the observed effects can be attributed to lipid–peptide interactions.

### Interaction of LL-37 Variants with Lipid Monolayers

**Figure 5:**
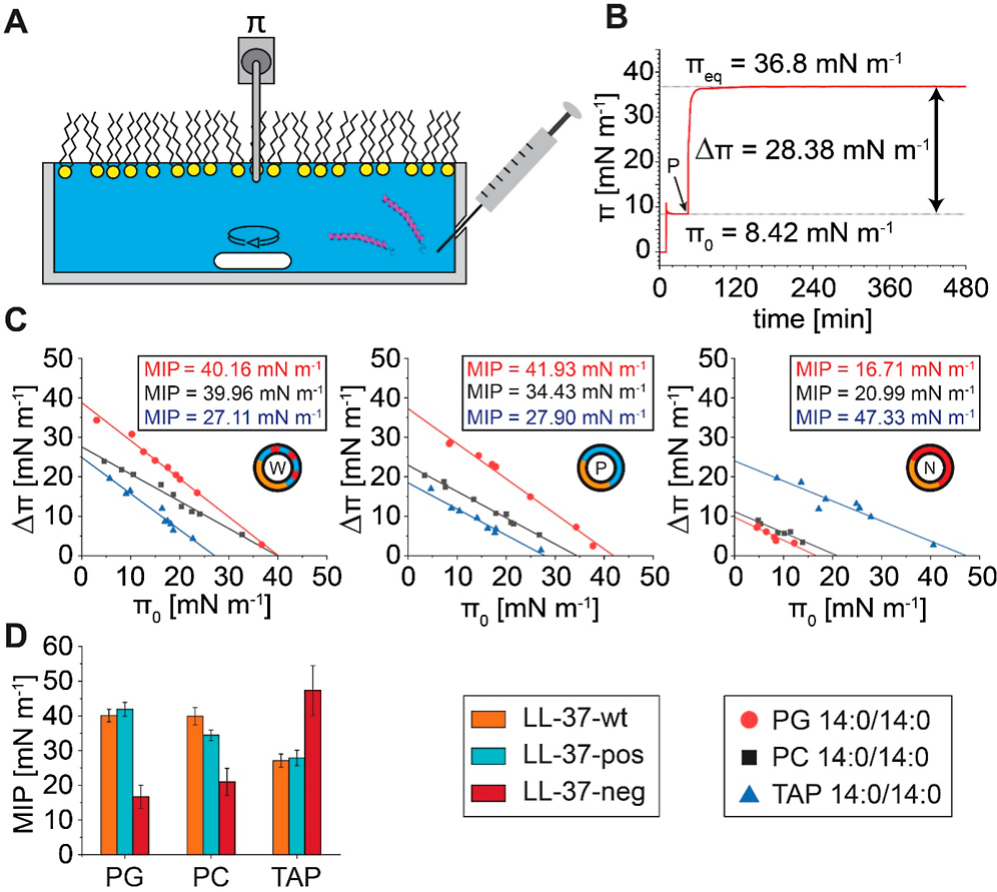
Exploring interactions of LL-37 variants with lipid monolayers. (A) Schematic of the experimental setup. Peptides were directly injected into a subphase (PBS) underneath the lipid monolayer. (B) The surface pressure was plotted against time for the insertion of 400 nM LL-37-pos into a PG 14:0/14:0 monolayer. The time point of peptide insertion (*P*), the initial surface pressure (π_0_), the increase in surface pressure (Δπ), and the equilibrium adsorption pressure (π_eq_) are given. (C) Δπ was plotted against π_0_ for the interaction of LL-37-wt (lhs), LL-37-pos (middle), and LL-37-neg (rhs) with PG 14:0/14:0 (red circles), PC 14:0/14:0 (black squares), and TAP 14:0/14:0 (blue triangles). Determined MIPs for the respective lipids are given in the insets. (D) The MIPs of LL-37-wt (orange), LL-37-pos (cyan), and LL-37-neg (red) for PG 14:0/14:0, PC 14:0/14:0, and TAP 14:0/14:0 monolayers are compared.

Having explored the surface activity of the three LL-37 variants, we proceeded to investigate their interactions with zwitterionic, negatively, and positively charged lipid monolayers. As the results obtained for native MS measurements were comparable between different zwitterionic or negatively charged lipids, we prepared three exemplary lipid monolayers using the negatively charged lipid PG 14:0/14:0, the zwitterionic lipid PC 14:0/14:0, and the cationic lipid analogue TAP 14:0/14:0. To study insertion of peptides, lipid monolayers were prepared at different surface pressures by spreading lipids dissolved in chloroform or chloroform/methanol mixtures at the air–water interface (Figure 5A). After injection of the peptide into the subphase underneath the lipid monolayer, we monitored the change in surface pressure caused by insertion of the peptides into the lipid monolayers. A peptide concentration of 400 nM (see above, Figure 4C) was selected for these experiments.

To characterize lipid preferences of the LL-37 variants in solution, the MIP was determined for different lipid monolayers. The MIP corresponds to the maximum surface pressure at which peptide insertion is energetically favorable.^29^ Notably, a π = 30 mN/m is commonly defined as the bilayer–monolayer equivalence pressure, i.e., the pressure at which the structure of a lipid monolayer resembles that of one phospholipid bilayer leaflet.^64−67^ Accordingly, if a MIP ≥ 30 mN/m is observed for a specific monolayer, the peptide inserts into a self-assembled bilayer of the same lipid. To determine the MIP values, we analyzed the change in surface pressure after peptide insertion (Δπ) at various initial surface pressures (π_0_) for the three different lipids in combination with each individual LL-37 variant (Figures 5B and S8). Next, we plotted Δπ as a function of π_0_ for each peptide–lipid combination (Figure 5C). The MIPs were obtained by extrapolating the linear regressions to determine the intercept with the *x* axis (see Section Experimental Methods).

For each LL-37 variant, the MIP values were extracted (Figure 5C) and compared (Figure 5D). For LL-37-wt, we observed a similar extent of penetration into PG 14:0/14:0 and PC 14:0/14:0 monolayers with MIPs of approximately 40 mN/m. These findings are in agreement with previous reports demonstrating similar binding and penetration of LL-37 into zwitterionic and anionic lipid membranes.^31^ In contrast, a lower MIP of approximately 27 mN/m was determined for the interaction with a TAP 14:0/14:0 monolayer, suggesting a preference for PG 14:0/14:0 and PC 14:0/14:0 over TAP 14:0/14:0. We postulate that these preferences are mainly defined by two factors: (i) Interactions with the negative charge of the phosphate group in PG 14:0/14:0 and PC 14:0/14:0 likely enhance the binding affinity of LL-37-wt. (ii) The position of the cationic functional group of TAP 14:0/14:0 relative to the hydrophilic binding interface of LL-37-wt is less favorable for the interactions. Accordingly, LL-37-wt was previously proposed to locate in the interfacial region between the lipid head groups and the hydrophobic core.^20^ Therefore, the interactions between PG 14:0/14:0 and PC 14:0/14:0 are similar, while interactions with TAP 14:0/14:0 are affected by the charge repulsion of LL-37-wt. In a similar fashion, several Gram-positive bacteria contain cationic lipid lysyl-phosphatidylglycerol in their outer layer membranes enhancing resistance against antimicrobial peptides via charge repulsion.^68^

For LL-37-pos with PG 14:0/14:0 monolayers, a MIP of approximately 42 mN/m was obtained. In contrast, MIPs for the interaction of LL-37-pos with PC 14:0/14:0 and TAP 14:0/14:0 were lower (approximately 34 and 28 mN/m, respectively). These findings indicate a higher selectivity of LL-37-pos for negatively charged lipids, likely caused by attractive interactions with the negatively charged PG 14:0/14:0 headgroup on the one hand and an increased charge repulsion between the positively charged amino acids and the positively charged choline groups of PC 14:0/14:0 and TAP 14:0/14:0 on the other hand. Note that MIP values obtained for LL-37-pos with PG 14:0/14:0 and TAP 14:0/14:0 monolayers are comparable to MIPs determined for LL-37-wt, while interactions with PC 14:0/14:0 are less favored for LL-37-pos than for LL-37-wt. Again, the enhanced charge repulsion of LL-37-pos might be the reason for this observation.

For LL-37-neg, low MIPs of approximately 17 and 21 mN/m were determined for the interactions with PG and PC monolayers, respectively. However, interactions of LL-37-neg with a TAP 14:0/14:0 monolayer resulted in the highest MIP of approximately 47 mN/m, indicating that LL-37-neg prefers cationic lipids over zwitterionic and negatively charged lipids. Considering a similar insertion mode of LL-37-neg into a the lipid membrane as described for LL-37-wt,^20^ interactions with negatively charged phosphate groups of PG 14:0/14:0 and PC 14:0/14:0 are less favored than with the positively charged functional group of TAP 14:0/14:0. Again, differences in the formation of the secondary structure might also affect the insertion of LL-37-neg and therefore the observed MIP values.

## Conclusions

In this study, we employed three LL-37 variants with different electrostatic properties to systematically investigate the impact of the surface charge of peptides on their interactions with different lipids in the gas phase and in solution. For this, we analyzed peptide–lipid interactions, formed through the transfer of lipids from mixed detergent–lipid micelles, by native MS in positive and negative ion modes. Making use of a film balance, we compared these results to interactions of the different peptide variants with lipid monolayers composed of exemplary lipids that were also used during native MS.

Native MS in positive ion mode revealed a preference of LL-37-wt and LL-37-pos for negatively charged lipids. A lower affinity was determined for PC 14:0/14:0, while interactions with a cationic lipid analogue (i.e., TAP 14:0/14:0) were not detected. Interestingly, in positive ion mode, interactions of LL-37-neg with all lipids employed in this study were of low abundance when compared with LL-37-wt and LL-37-pos. In contrast, when using negative ion mode, LL-37-neg showed a higher affinity for the cationic lipid TAP 14:0/14:0, a comparably low affinity for the zwitterionic lipid PC 14:0/14:0 and no affinity for the negatively charged lipid PG 14:0/14:0. Adsorption measurements using a Langmuir film balance revealed similar binding preferences of LL-37-wt and LL-37-pos for PG 14:0/14:0 and PC 14:0/14:0 as well as low binding affinity for TAP 14:0/14:0. Similar to native MS, LL-37-neg showed a low binding affinity for PG 14:0/14:0 and PC 14:0/14:0 (as observed in positive ion mode) and a high binding affinity for TAP 14:0/14:0 (as observed in negative ion mode).

Comparing the lipid preferences of LL-37-wt and LL-37-pos determined by native MS and by Langmuir film balance, the overall trend observed by both methods is similar, with the only exception that interactions of LL-37-wt with PC 14:0/14:0 were higher with a Langmuir film balance. For LL-37-neg, only lipid preferences determined in negative ion mode correlate with the preferences determined in solution, indicating that the positive ion mode is not suited for the analysis of interactions between peptides with negative solution charge and their ligands. Note that the interactions of LL-37-neg with PG 14:0/14:0 might also be underestimated because the ionization of PG 14:0/14:0 is more efficient in negative ion mode. In agreement, interactions with PG 14:0/14:0 and PC 14:0/14:0 monolayers resulted in low MIPs around 20 mN/m, while interactions with TAP 14:0/14:0 resulted in a high MIP (47 mN/m).

Our findings are in agreement with previous studies showing that LL-37 is sensitive to the composition of the target membranes. For instance, a bacterial defense mechanism against LL-37 includes the expression of untypical phosphorylcholine modulating LL-37–membrane interactions and decreasing the antimicrobial activity of the peptide.^69^ Furthermore, aggregation of LL-37 is higher in zwitterionic PC membranes when compared with negatively charged PC/PS membranes.^31^ Accordingly, both studies confirm that the preferred natural membrane environment of LL-37 includes negatively charged phospholipids.

From a technical point of view, although relative abundances of complexes identified in the gas phase by native MS and MIPs determined through adsorption at lipid monolayers using a Langmuir film balance cannot be directly compared, both approaches reveal potential differences in the electrostatic interactions of peptides and lipids. The relative complex abundances and MIP values correlate well for the interactions of cationic peptides LL-37-wt and LL-37-pos. Importantly, a correlation of gas phase and solution interactions was also observed for LL-37-neg, however, only when employing the negative ion mode. Interestingly, discrepancies were identified for interactions of the two variants LL-37-wt and LL-37-pos with the zwitterionic lipid PC 14:0/14:0. We assume that, in addition to potentially different binding interfaces between peptides and lipids in these two approaches, ionization effects might lead to an underestimation of complex formation containing cationic peptides and positively charged ligands in positive ion mode. Our findings underline the need for uncovering the mechanism of lipid transfer from detergent–lipid micelles; this procedure appears to be influenced by the ionization mechanism during native MS and potentially the binding interface between the peptide and the ligand. Nonetheless, we demonstrate the capability of native MS for determining the general binding preferences of peptides; however, the ion mode influences the observed interactions and should be selected with care. Accordingly, the positive ion mode is applicable for the analysis of cationic peptides, while the analysis of negatively charged peptides might require the negative ion mode.

In summary, our comparison reveals, for both approaches, strong effects of electrostatic interactions on peptide–lipid interactions. Accordingly, the highly charged peptide variants (i.e., LL-37-pos and LL-37-neg) show preferences for lipids with opposite charge, while the wild-type variant contains positively charged and negatively charged amino acid residues and, therefore, is more flexible in the formation of interactions with various (phospho-) lipids. The effects of the structural content as well as structure formation during membrane insertion remain to be elucidated in future experiments. Importantly, our study together with other previous and potential future studies further contributes to the general understanding of peptide–membrane interactions. These findings will therefore be of interest for different lines of research, for instance, in pharmaceutical applications designing artificial antimicrobial peptides.

## Supporting Information Available

Figures of structures of lipids employed in this study, effects of C8E4 on the ionization properties of the LL-37 variants, DLS analysis of detergent–lipid micelles, exploration of electrostatic interactions between the LL-37 variants and negatively charged lipids, explorarion of interactions between the LL-37 variants and zwitterionic and positively charged lipids, exploration of interactions between LL-37-neg and lipids in negative ion mode, investigation of surface activity of the LL-37 variants by adsorption film balance, and interactions of the LL-37 variants with lipid monolayers studied by adsorption film balance and tables of properties of the used LL-37 variants and masses of LL-37 variants and peptide–lipid complexes determined by native MS (PDF)
