# Supplementary material for: Effects of Surface Charge of Amphiphilic Peptides on Peptide–Lipid Interactions in the Gas Phase and in Solution
Source: Anal Chem. 2025 Mar 7;97(10):5808–17. doi: 10.1021/acs.analchem.5c00283 (PMC11923945; doi:10.1021/acs.analchem.5c00283)
Supplement: Supplementary file 1 — ac5c00283_si_001.pdf [file ac5c00283_si_001.pdf]

Supporting Information

On the effects of surface charge of amphiphilic peptides on peptide-lipid interactions in the gas phase and in solution

Til Kundlacz[1], Christian Schwieger[1], Carla Schmidt[2*]

[1]Institute of Chemistry, Martin Luther University Halle-Wittenberg, von-Danckelmann-Platz 4, 06120 Halle, Germany

[2]Johannes Gutenberg-University Mainz, Department of Chemistry – Biochemistry, Biocenter II, Hanns-Dieter-Hüsch-Weg 17, 55128 Mainz, Germany

*correspondence: carla.schmidt@uni-mainz.de

Table of Contents Page

Supporting Figures 3

Figure S1: Structures of the lipids employed in this study. 3

Figure S2: Effects of C8E4 on the ionisation properties of the LL-37 variants. 4

Figure S3: DLS analysis of detergent-lipid micelles. 5

Figure S4: Exploring electrostatic interactions between the LL-37 variants and negatively charged lipids. 6

Figure S5: Exploring interactions between the LL-37 variants and zwitterionic and positively charged lipids. 7

Figure S6: Exploring interactions between LL-37-neg and lipids in negative ion mode. 8

Figure S7: Investigating the surface activity of the LL-37 variants by adsorption film balance. 9

Figure S8: Interactions of the LL-37 variants with lipid monolayers studied by adsorption film balance. 10

Supporting Tables 11

Table S1: Properties of the used LL-37 variants. 11

Table S2: Masses of LL-37 variants and peptide-lipid complexes determined by native MS. 12

References .... **Fehler! Textmarke nicht definiert.**

Supporting Figures

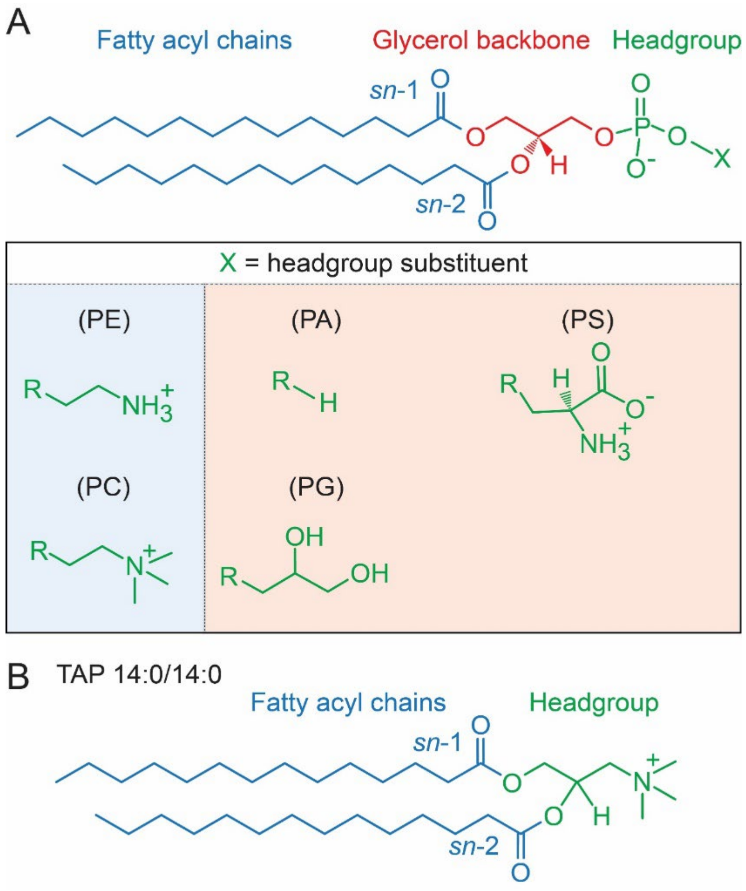

Figure S1: Structures of the lipids employed in this study. (A) Structures of the different phospholipids. Zwitterionic (blue) and negatively charged (red) head group substituents are given. **(B)** Structure of the positively charged lipid analogue TAP 14:0/14:0. Figure adapted from Harayama et al. 2018[1].

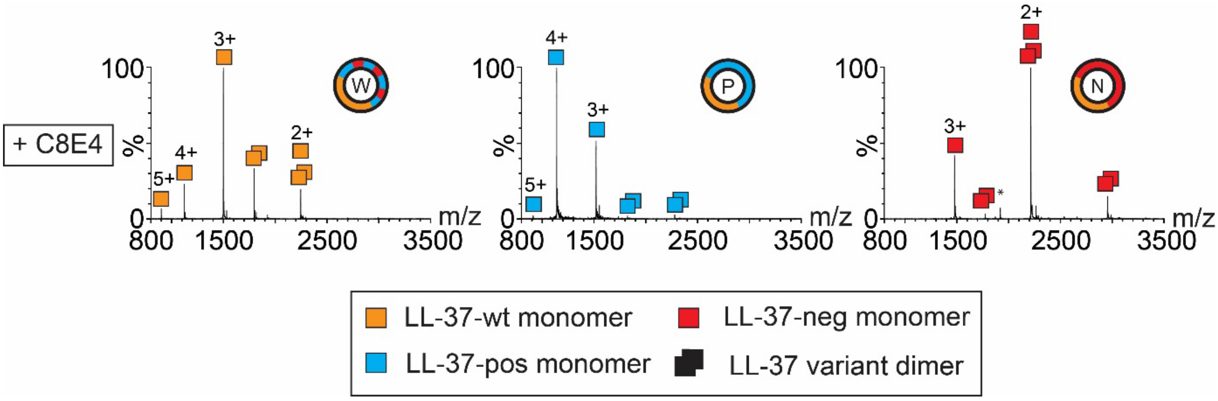

Figure S2: Effects of C8E4 on the ionisation properties of the LL-37 variants. Native MS of 20 µM LL-37-wt (left), LL-37-pos (middle) and LL-37-neg (right) in the presence of 0.5% C8E4. Charge states and lipid adducts of the respective monomer species are assigned. Due to the activation energy required for native MS analysis, a low-intense fragment ion of the C-terminal coil region of LL-37-neg was observed (marked by asterisk). Masses of the peptide-lipid complexes are given in **Table S2.**

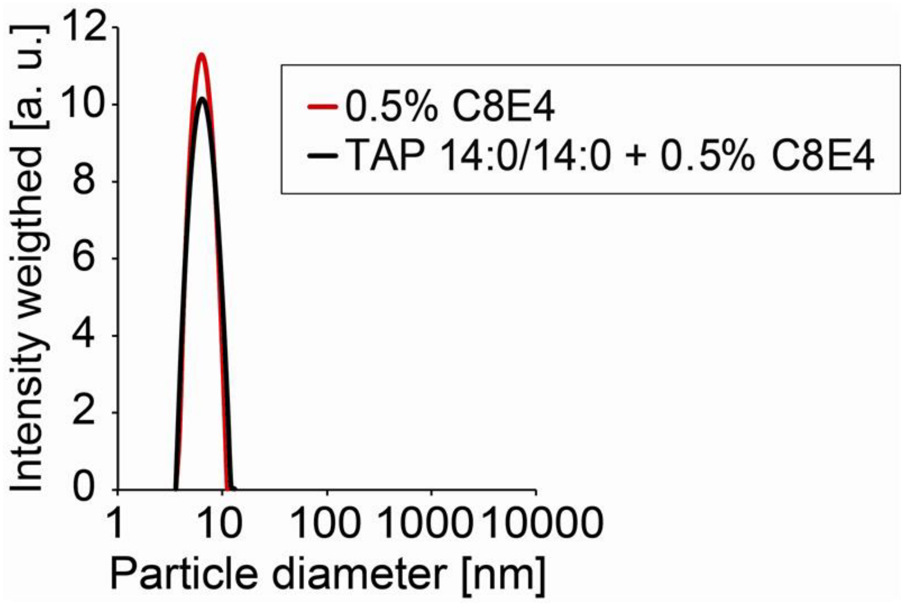

Figure S3: **DLS analysis of detergent-lipid micelles.** Intensity weighted particle size distribution of C8E4 micelles and C8E4-lipid micelles containing TAP 14:0/14:0.

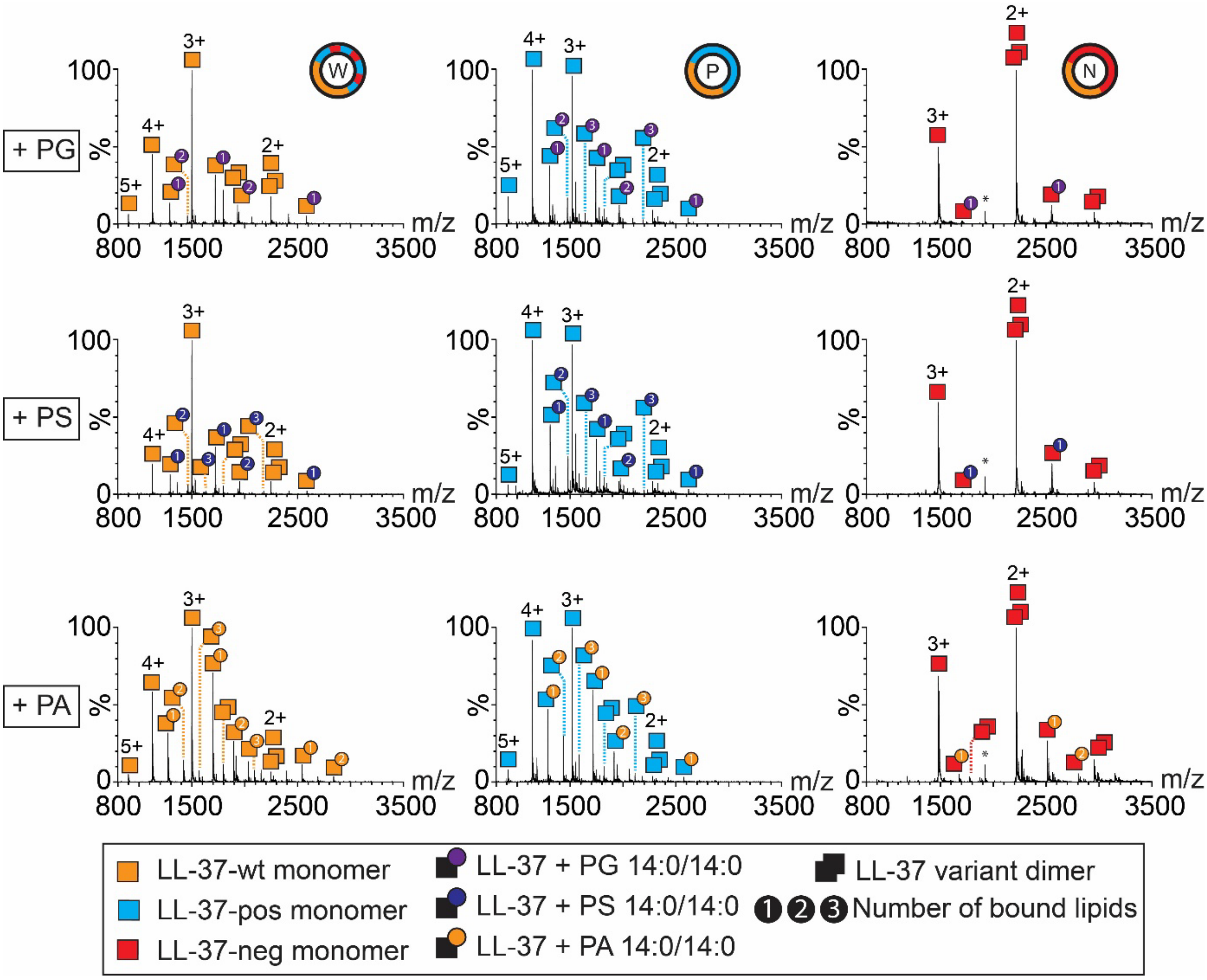

Figure S4: **Exploring electrostatic interactions between the LL-37 variants and negatively charged lipids.** Native MS of 20 µM LL-37-wt (left), LL-37-pos (middle) and LL-37-neg (right) in the presence of 0.5 % (w/v) C8E4 and after addition of 25 µM PG 14:0/14:0 (purple), PS 14:0/14:0 (blue) and PA 14:0/14:0 (orange). Charge states and lipid adducts of the respective monomer species are assigned. A fragment ion of the C-terminal coil region of LL-37-neg was observed (marked by asterisk) Masses of the peptide-lipid complexes are given in **Table S2.**

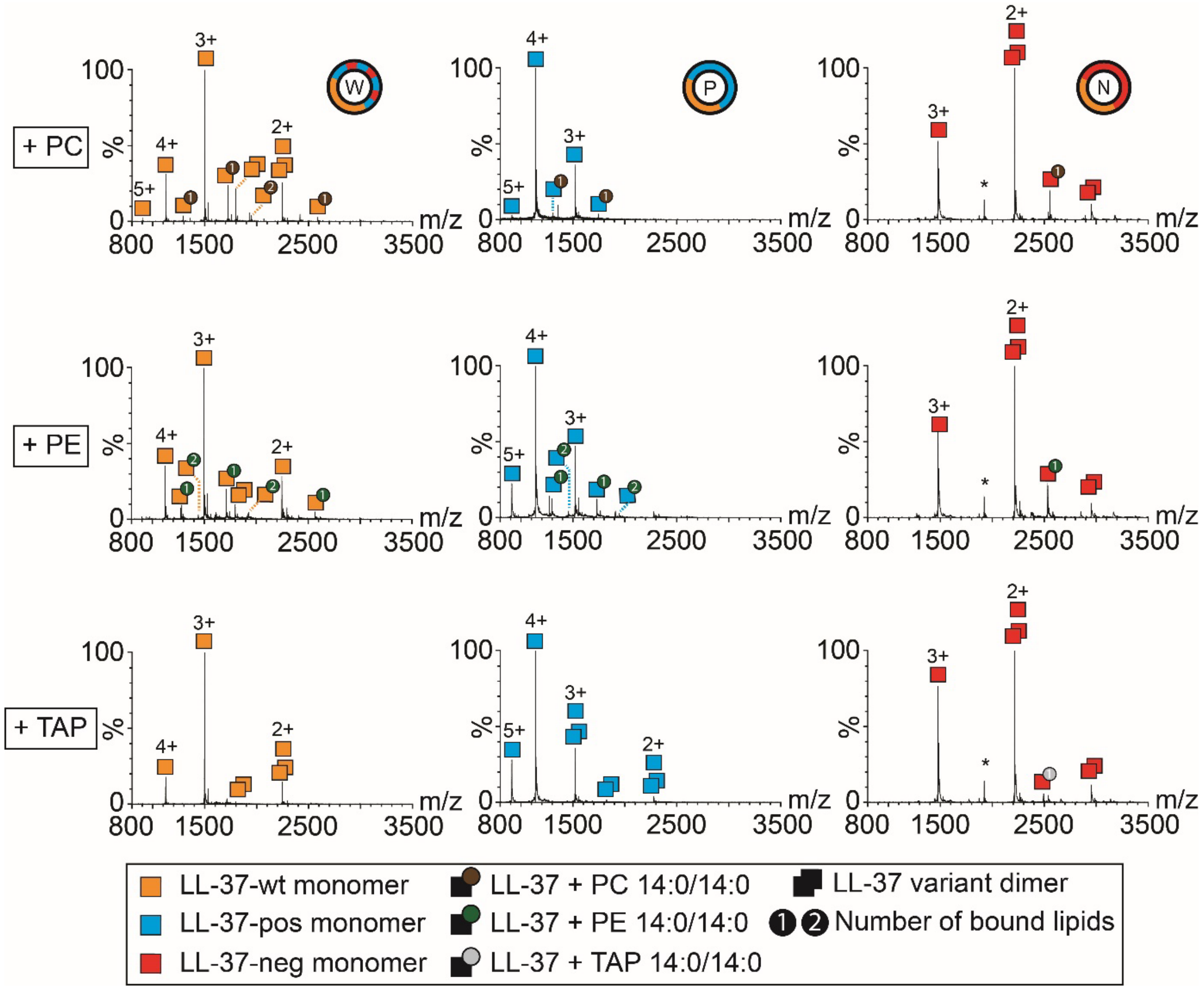

Figure S5: **Exploring interactions between the LL-37 variants and zwitterionic and positively charged lipids.** Native MS of 20 µM LL-37-wt (left), LL-37-pos (middle) and LL-37-neg (right) in the presence of 0.5 % (w/v) C8E4 as well as 25 µM PC 14:0/14:0 (brown), PE 14:0/14:0 (green) or TAP 14:0/14:0 (grey). Charge states and lipid adducts of the respective monomer species are assigned. Fragmentation of a small population of LL-37-neg in its C-terminal coil region was observed (marked by asterisk). Masses of the peptide-lipid complexes are given in **Table S2.**

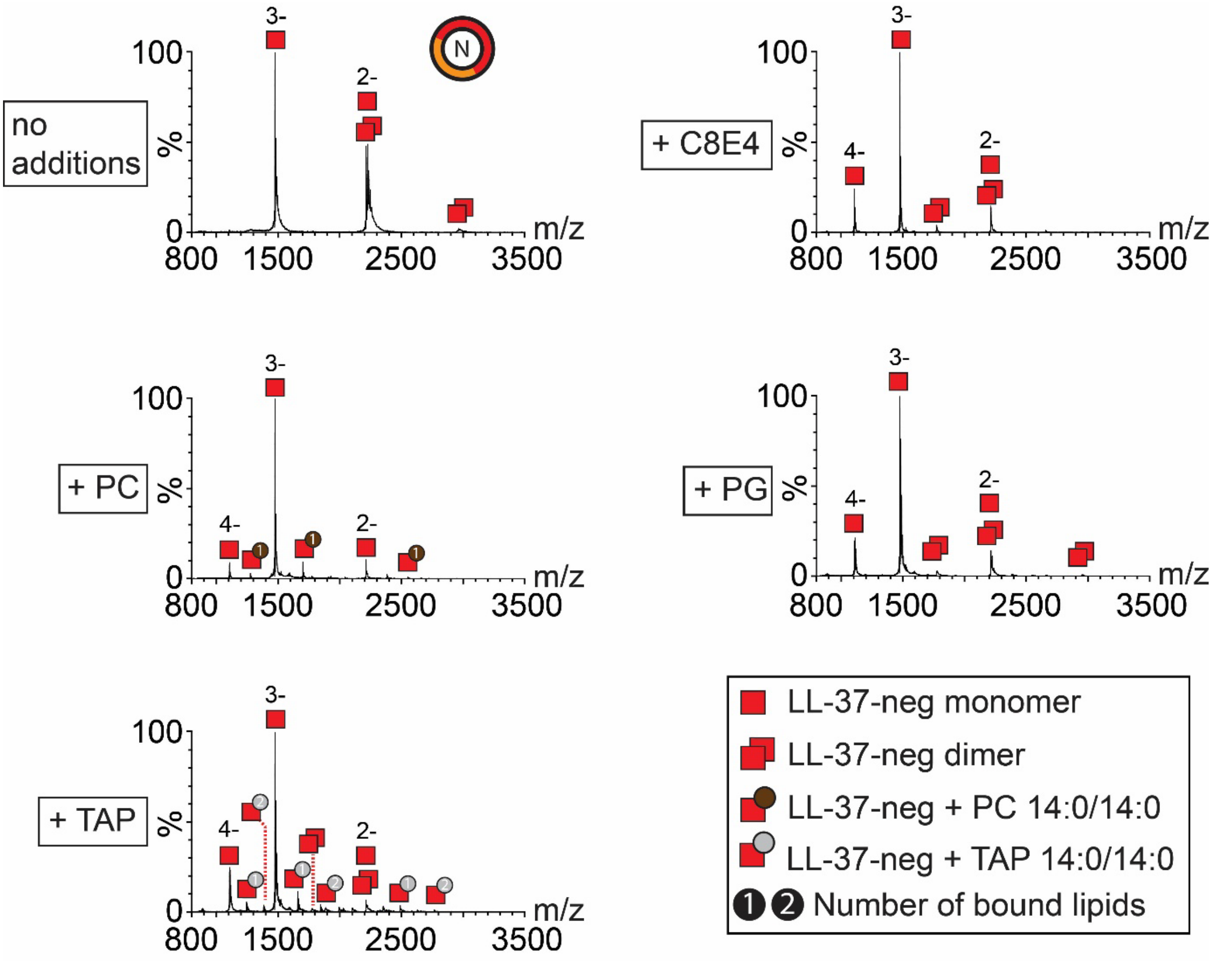

Figure S6: **Exploring interactions between LL-37-neg and lipids in negative ion mode.** Native mass spectra of 10 µM LL-37-neg in 200 mM ammonium acetate in the absence and presence of 0.5 % (w/v) C8E4 as well as with 25 µM PC 14:0/14:0 (brown), 25 µM PG (no binding) and 25 µM TAP 14:0/14:0 (grey). Charge states and lipid adducts of the LL-37-neg monomers are assigned. Masses of the peptide-lipid complexes are given in **Table S2.**

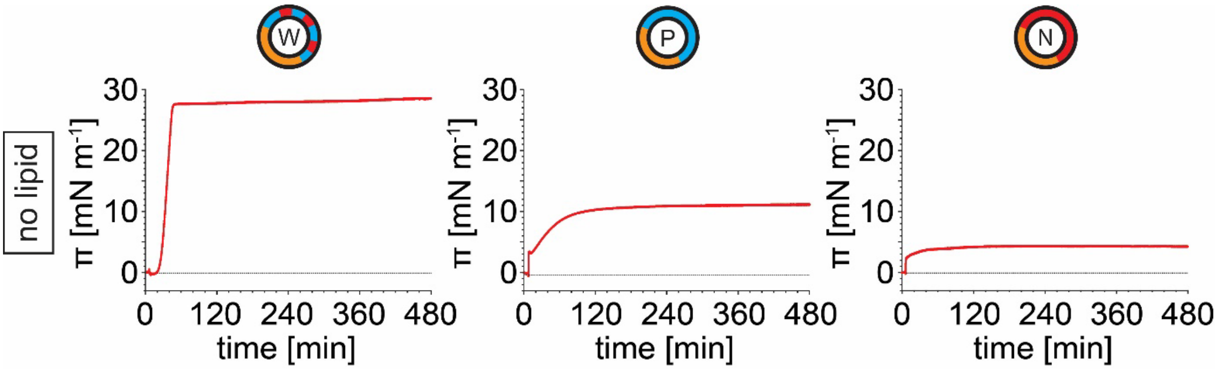

Figure S7: **Investigating the surface activity of the LL-37 variants by adsorption film balance.** Surface pressure versus time diagram for the adsorption of 250 nM LL-37-wt (left), 75 nM LL-37-pos (middle) and 50 nM LL-37-neg (right) at the air-water interface. Peptide was injected after incubation of the subphase for approx. 10 min (see **Methods**). The initial surface pressure π_0 is given by the dashed line.

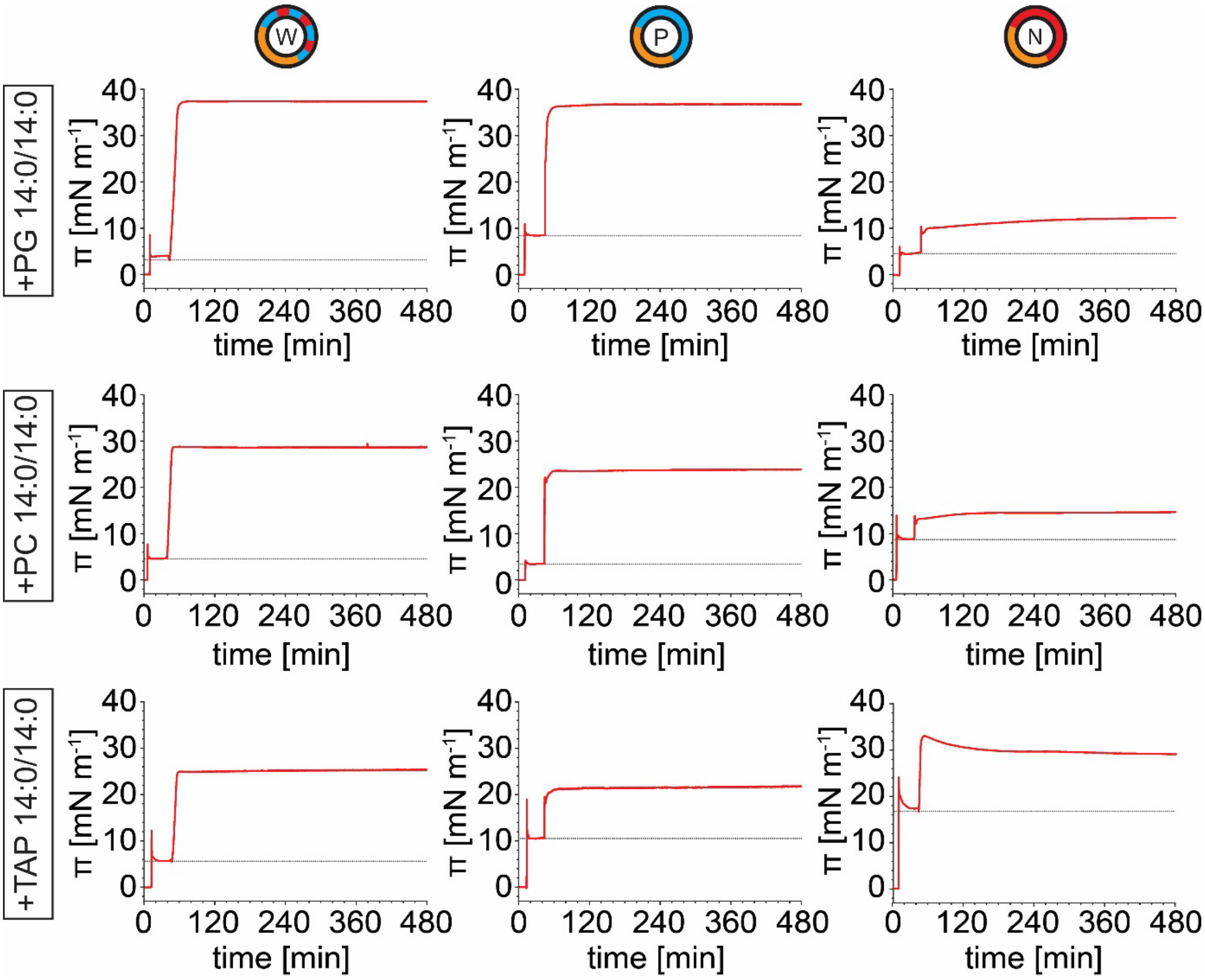

Figure S8: **Interactions of the LL-37 variants with lipid monolayers studied by adsorption film balance.** The surface pressure was plotted against time for the interactions of 400 nM LL-37-wt (left), LL-37-pos (middle) and LL-37-neg (right) with selected PG 14:0/14:0 (top), PC 14:0/14:0 (middle) and TAP 14:0/14:0 (bottom) lipid monolayers. After incubation of the subphase for approx. 10 min, lipids dissolved in chloroform or a chloroform/methanol mixture were spread at the air-water interface until the desired surface pressure is reached. The resulting lipid monolayers were then incubated for approx. 30 min before the respective peptides were injected underneath the monolayer (see **Methods** for details). The initial surface pressure π_0 is indicated by the dashed line.

Supporting Tables

Table S1: Properties of the used LL-37 variants. The molecular weight of the non-charged peptides, the theoretical pI and the grand average hydropathicity (GRAVY) were calculated using ProtParam[2]. The GRAVY index indicates the hydrophobicity of an amino acid sequence calculated through hydropathy values by Kyte and Doolittle[3] with higher values indicating higher hydrophobicity.

Criteria	LL-37-wt	LL-37-pos	LL-37-neg
Amino acid sequence	LLGDFFRKSKEKIGKEFKRIVQRIKDFLRNLV PRTES	LLGKFFRKSKKKIGKKWKRIVQRIKKFLRNLV PRTES	LLGDFFEESEEEIGEEWEEIVQEIEDFLENLV PRTES
Symbol	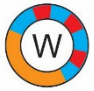	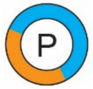	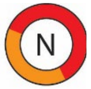
Molecular weight [Da]	4493.32	4556.65	4429.72
Theoretical pI	10.61	12.20	3.44
Basic amino acids	11	15	1
Acidic amino acids	5	1	15
Solution net charge	6+	14+	14-
Grand average of hydropathicity (GRAVY)	-0.724	-0.868	-0.651

Table S2: **Masses of LL-37 variants and peptide-lipid complexes determined by native MS.** The figure, the additives, the respective LL-37 variant / LL-37-lipid complex, the theoretical mass and the experimental mass of assigned peaks are given. Theoretical masses were provided by the peptide and lipid manufacturer. Experimental masses and the corresponding error were determined using MassLynx v4.1.

Figure	Additives	LL-37 variant/ LL-37-lipid complex	Theoretical mass (Da)	Experimental mass (Da)
1	none	LL-37-wt monomer	4492.28	4492.55 ± 0.00
		LL-37-wt dimer	8984.56	8984.91 ± 0.19
		LL-37-pos monomer	4556.66	4556.71 ± 0.00
		LL-37-neg monomer	4429.74	4429.74 ± 0.33
		LL-37-neg monomer adduct (peak with highest intensity)	/	4446.35 ± 0.28
		LL-37-neg dimer	8858.44	8858.66 ± 0.28
S1	0.5 % (w/v) C8E4	LL-37-wt monomer	4492.28	4492.19 ± 0.07
		LL-37-wt dimer	8984.56	8983.83 ± 0.04
	0.5 % (w/v) C8E4	LL-37-pos monomer	4556.66	4555.86 ± 0.09
		LL-37-pos dimer	9113.32	9112.93 ± 0.25
	0.5 % (w/v) C8E4	LL-37-neg monomer	4429.74	4428.9 ± 0.00
		LL-37-neg dimer	8859.48	8858.39 ± 0.00
S3	0.5 % (w/v) C8E4 + PG 14:0/14:0	LL-37-wt monomer	4492.28	4491.57 ± 0.02
		LL-37-wt dimer	8984.56	8984.62 ± 0.16
		LL-37-wt + 1 × PG 14:0/14:0	5158.125	5159.04 ± 0.01
		LL-37-wt + 2 × PG 14:0/14:0	5823.97	5825.92 ±0.00
	0.5 % (w/v) C8E4 + PS 14:0/14:0	LL-37-wt monomer	4492.28	4491.67 ± 0.06
		LL-37-wt dimer	8984.56	8984.54 ± 0.20
		LL-37-wt + 1 × PS 14:0/14:0	5171.124	5171.61 ± 0.40
		LL-37-wt + 2 × PS 14:0/14:0	5849.968	5850.79 ± 0.10
		LL-37-wt + 3 × PS 14:0/14:0	6528.812	6531.41 ± 0.00
	0.5 % (w/v) C8E4 + PA14:0/14:0	LL-37-wt monomer	4492.28	4491.68 ± 0.14
		LL-37-wt dimer	8984.56	8984.40 ± 0.65
		LL-37-wt + 1 × PA 14:0/14:0	5084.047	5084.91 ± 0.00
		LL-37-wt + 2 × PA 14:0/14:0	5675.814	5676.94 ± 0.00
		LL-37-wt + 3 × PA 14:0/14:0	6267.581	6270.19 ± 0.18
	0.5 % (w/v) C8E4 + PG 14:0/14:0	LL-37-pos monomer	4556.66	4556.42± 0.39
		LL-37-pos dimer	9113.32	9113.07 ± 0.29
		LL-37-pos + 1 × PG 14:0/14:0	5222.505	5223.05 ± 0.21

		LL-37-pos + 2 × PG 14:0/14:0	5888.35	5889.97 ± 0.09
		LL-37-pos + 3 × PG 14:0/14:0	6554.195	6556.30 ± 0.09
	0.5 % (w/v) C8E4 + PS 14:0/14:0	LL-37-pos monomer	4556.66	4555.90± 0.07
		LL-37-pos dimer	9113.32	9112.55 ± 0.41
		LL-37-pos + 1 × PS 14:0/14:0	5235.504	5236.03 ± 0.23
		LL-37-pos + 2 × PS 14:0/14:0	5914.348	5916.17 ± 0.15
		LL-37-pos + 3 × PS 14:0/14:0	6593.192	6595.25 ± 0.09
	0.5 % (w/v) C8E4 + PA 14:0/14:0	LL-37-pos monomer	4556.66	4555.87 ± 0.01
		LL-37-pos dimer	9113.32	9113.40 ± 0.11
		LL-37-pos + 1 × PA 14:0/14:0	5148.427	5149.23 ± 0.02
		LL-37-pos + 2 × PA 14:0/14:0	5740.194	5741.76 ± 0.05
		LL-37-pos + 3 × PA 14:0/14:0	6331.961	6334.45 ± 0.40
	0.5 % (w/v) C8E4 + PG 14:0/14:0	LL-37-neg monomer	4429.74	4429.21 ± 0.00
		LL-37-neg dimer	8858.44	8858.93 ± 0.45
		LL-37-neg + 1 × PG 14:0/14:0	5095.585	5096.53 ± 0.13
	0.5 % (w/v) C8E4 + PS 14:0/14:0	LL-37-neg monomer	4429.74	4429.39 ± 0.02
		LL-37-neg dimer	8858.44	8859.09 ± 0.00
		LL-37-neg + 1 × PS 14:0/14:0	5108.584	5109.13 ± 0.05
	0.5 % (w/v) C8E4 + PA 14:0/14:0	LL-37-neg monomer	4429.74	4429.22 ± 0.00
		LL-37-neg dimer	8858.44	8859.50 ± 0.00
		LL-37-neg + 1 × PA 14:0/14:0	5021.507	5021.73 ± 0.01
S4	0.5 % (w/v) C8E4 + PC 14:0/14:0	LL-37-wt monomer	4492.28	4491.51 ± 0.00
		LL-37-wt dimer	8984.56	8984.08 ± 0.08
		LL-37-wt + 1 × PC 14:0/14:0	5170.23	5170.08± 0.05
		LL-37-wt + 2 × PC 14:0/14:0	5848.18	5849.01 ± 0.05
	0.5 % (w/v) C8E4 + PE 14:0/14:0	LL-37-wt monomer	4492.28	4491.25 ± 0.03
		LL-37-wt dimer	8984.56	8982.71 ± 0.39
		LL-37-wt + 1 × PE 14:0/14:0	5128.133	5127.66± 0.10
		LL-37-wt + 2 × PE 14:0/14:0	5763.986	5762.78 ± 0.22
	0.5 % (w/v) C8E4 + TAP 14:0/14:0	LL-37-wt monomer	4492.28	4492.04 ± 0.03
		LL-37-wt dimer	8984.56	8984.07 ± 0.10
	0.5 % (w/v) C8E4 + PC 14:0/14:0	LL-37-pos monomer	4556.66	4556.12 ± 0.26
		LL-37-pos + 1 × PC 14:0/14:0	5234.61	5233.43 ± 0.00
	0.5 % (w/v) C8E4 + PE 14:0/14:0	LL-37-pos monomer	4556.66	4556.71 ± 0.01
		LL-37-pos + 1 × PE 14:0/14:0	5192.513	5192.65 ± 0.05

		LL-37-pos + 2 × PE 14:0/14:0	5828.366	5828.80 ± 0.05
	0.5 % (w/v) C8E4 + TAP 14:0/14:0	LL-37-pos monomer	4556.66	4556.63 ± 0.04
		LL-37-pos dimer	9113.320	9113.320 ± 0.04
	0.5 % (w/v) C8E4 + PC 14:0/14:0	LL-37-neg monomer	4429.74	4429.41 ± 0.10
		LL-37-neg dimer	8858.44	8859.56 ± 0.02
		LL-37-neg 1 × PC 14:0/14:0	5107.69	5106.73 ± 0.09
	0.5 % (w/v) C8E4 + PE 14:0/14:0	LL-37-neg monomer	4429.74	4429.41 ± 0.10
		LL-37-neg dimer	8858.44	8859.05 ± 0.02
		LL-37-neg 1 × PE 14:0/14:0	5065.59	5065.76 ± 0.00
	0.5 % (w/v) C8E4 + TAP 14:0/14:0	LL-37-neg monomer	4429.74	4429.80 ± 0.00
		LL-37-neg dimer	8858.44	8859.63 ± 0.03
		LL-37-neg 1 × TAP 14:0/14:0	4984.724	4983.66 ± 0.01
S5	none	LL-37-neg monomer	4429.74	4429.80 ± 0.11
		LL-37-neg dimer	8858.44	8860.12 ± 0.02
	0.5 % (w/v) C8E4	LL-37-neg monomer	4429.74	4431.49 ± 0.00
		LL-37-neg dimer	8858.44	8867.17 ± 4.19
	0.5 % (w/v) C8E4 + PC 14:0/14:0	LL-37-neg monomer	4429.74	4429.41 ± 0.10
		LL-37-neg 1 × PC 14:0/14:0	5107.69	5107.34 ± 0.09
	0.5 % (w/v) C8E4 + PG 14:0/14:0	LL-37-neg monomer	4429.74	4429.65 ± 0.00
		LL-37-neg dimer	8858.44	8859.25 ± 0.06
	0.5 % (w/v) C8E4 + TAP 14:0/14:0	LL-37-neg monomer	4429.74	4429.48 ± 0.05
		LL-37-neg dimer	8858.44	8865.25 ± 6.19
		LL-37-neg + 1 × TAP 14:0/14:0	4984.724	4983.20 ± 0.0
		LL-37-neg + 2 × TAP 14:0/14:0	5539.708	5536.96 ± 0.00

References

(1) Harayama, T.; Riezman, H. Understanding the diversity of membrane lipid composition. *Nature reviews. Molecular cell biology* **2018**, *19*, 281–296.
(2) Gasteiger, E.; Hoogland, C.; Gattiker, A.; Duvaud, S.'e.; Wilkins, M. R.; Appel, R. D.; Bairoch, A. In The Proteomics Protocols Handbook; Walker, John M., Ed.; Humana Press: Totowa, NJ, 2005; pp. 571–607.
(3) Kyte, J.; Doolittle, R. F. A simple method for displaying the hydropathic character of a protein. *Journal of molecular biology* **1982**, *157*, 105–132.
